# Association of spermidine blood levels with microstructure of sleep—implications from a population-based study

**DOI:** 10.1007/s11357-023-00886-3

**Published:** 2023-08-07

**Authors:** Silke M. Wortha, Juliane Schulz, Jevri Hanna, Claudia Schwarz, Beate Stubbe, Stefan Frenzel, Robin Bülow, Nele Friedrich, Matthias Nauck, Henry Völzke, Ralf Ewert, Antje Vogelgesang, Hans J. Grabe, Julia Ladenbauer, Agnes Flöel

**Affiliations:** 1 Department of Neurology, University Medicine Greifswald, Greifswald, Germany; 2 Centre for Mathematical Cognition, School of Science, Loughborough University, Loughborough, UK; 3 Department of Internal Medicine B, University Medicine Greifswald, Greifswald, Germany; 4 Department of Psychiatry and Psychotherapy, University Medicine Greifswald, Greifswald, Germany; 5 Institute for Diagnostic Radiology and Neuroradiology, University Medicine Greifswald, Greifswald, Germany; 6 Institute of Clinical Chemistry and Laboratory Medicine, University Medicine Greifswald, Greifswald, Germany; 7 German Centre for Cardiovascular Research (DZHK), Partner Site Greifswald, Greifswald, Germany; 8 Institute for Community Medicine, University Medicine Greifswald, Greifswald, Germany; 9 German Centre for Neurodegenerative Diseases (DZNE), Site Rostock/Greifswald, Greifswald, Germany

**Keywords:** Spermidine, Slow wave sleep, Alzheimer’s disease, Sleep, Brain health

## Abstract

Deteriorations in slow wave sleep (SWS) have been linked to brain aging and Alzheimer’s disease (AD), possibly due to its key role in clearance of amyloid-beta and tau (Aß/tau), two pathogenic hallmarks of AD. Spermidine administration has been shown to improve sleep quality in animal models. So far, the association between spermidine levels in humans and parameters of SWS physiology are unknown but may be valuable for therapeutic strategies. Data from 216 participants (age range 50–81 years) of the population-based Study of Health in Pomerania TREND were included in our analysis. We investigated associations between spermidine plasma levels, key parameters of sleep macroarchitecture and microarchitecture that were previously associated with AD pathology, and brain health measured via a marker of structural brain atrophy (AD score). Higher spermidine levels were significantly associated with lower coupling between slow oscillations and spindle activity. No association was evident for SWS, slow oscillatory, and spindle activity throughout non-rapid eye movement sleep. Furthermore, elevated spermidine blood levels were significantly associated with a higher AD score, while sleep markers revealed no association with AD score. The association between higher spermidine levels and brain health was not mediated by coupling between slow oscillations and spindle activity. We report that higher spermidine blood levels are associated not only with deteriorated brain health but also with less advantageous markers of sleep quality in older adults. Future studies need to evaluate whether sleep, spermidine, and Aß/tau deposition are interrelated and whether sleep may play a mediating role.

**Supplementary Information:**

The online version contains supplementary material available at 10.1007/s11357-023-00886-3.

## Introduction

### Aging, neurodegeneration, and prevention

Population-wise, aging is associated with deteriorating brain health [1]. Thus, in an aging society, the prevalence of neurodegenerative diseases such as Alzheimer’s disease (AD) is steadily increasing [2]. Consequently, it is of utmost importance to identify predictors and risk factors, and to investigate how they might interrelate, to optimally develop preventive and therapeutic strategies counteracting deterioration in brain health.

### Brain health and sleep

One important predictor for brain health in aging is sleep [3–5]. Growing evidence indicates a bidirectional interaction between sleep and pathophysiology, especially related to AD [6]. Sleep is implicated in the clearance of metabolic waste products from the brain that accumulate during wakefulness, such as Aβ [7] and tau [8], two major hallmarks of AD [9].

Regarding amyloid-beta (Aß) clearance, there is substantial evidence from animal as well as human studies indicating the major role of sleep in Aß clearance [7, 10–13]. More recent studies link this biological process particularly to SWS and the expression of slow oscillatory electrophysiological rhythms < 1 Hz (slow oscillation (SO)) [10–12, 14].

Similarly, increased tau burden and tau hyperphosphorylation have been linked to poor sleep quality and chronic sleep deprivation [8, 15–18]. Interestingly, spindles and their coupling with SO events, rather than slow wave activity, have been associated with tau deposition [19]. In summary, age-related decline of key parameters of SWS might be causally involved in accelerated brain aging and AD-related brain changes.

### Spermidine and brain health

One factor that has been suggested to have a positive impact on brain health is spermidine supplementation. Several animal and human studies have investigated the role of spermidine for healthy aging. Here, animal models suggested that spermidine supports healthy aging because of its benefits on the cardiovascular system, the immune system, and brain structure and function [20]. Evidence for the role of spermidine in structural brain health in human studies is mixed. A recent cross-sectional study found a positive relationship between dietary spermidine intake and hippocampal volume, as well as cortical thickness [21]. However, Tavares et al. [22] found that higher dietary spermidine intake was associated with smaller cortical and left hippocampal volume. In addition, several post-mortem studies could show that polyamine tissue levels were elevated in frontal and occipital lobes [23] as well as in temporal cortex, white matter, and thalamus in patients with AD [24, 25]. In line, we recently demonstrated that higher spermidine blood levels were significantly associated with advanced brain aging and deteriorated brain health indicated by lower hippocampal volume, higher AD score (greater AD related atrophy), and lower global cortical thickness [26]. In sum, despite previously reported beneficial effects of spermidine supplementation and dietary spermidine intake in animal models and human studies, neither blood nor tissue levels of spermidine have shown consistent positive associations with brain health in human studies.

### Spermidine and sleep

Previous animal models started to shed some light on the role of spermidine metabolism in sleep homeostasis and circadian rhythm [27, 28]. For instance, Zwighaft et al. [27] could show that age-related decline of polyamine levels in mice was associated with longer circadian periods. Moreover, this finding was reversable with spermidine supplementation. In addition, supplementation with spermidine in the fruit fly *Drosophila melanogaster* improved age-related detrimental changes in sleep physiology [29]. Although these studies indicate a role for spermidine on sleep in animal models, the relationship between spermidine and sleep and their interplay with brain health in humans is yet unknown.

### Open questions

Therefore, the present study aimed to evaluate whether and how spermidine plasma levels, SWS physiology, and brain health are interrelated in aging. We addressed these questions by first investigating associations between spermidine plasma levels and sleep physiology in humans using data from a large-scale cross-sectional epidemiological cohort [30]. We particularly focused on parameters of sleep macrostructure and microstructure that were shown to be associated with core pathological features of AD. Second, we evaluated the associations between spermidine blood levels, sleep, and brain health operationalized by grey and white matter integrity related to advanced brain aging via the AD score. Finally, we were interested in potentially mediating effects of SWS physiology on the association between spermidine and AD score.

## Methods

### Study sample

The Study of Health in Pomerania (SHIP) was designed to determine the prevalence of common risk factors and diseases in a population in northeastern Germany [31]. For this purpose, a sample was randomly drawn from local registers. Between 1997 and 2001, 4308 individuals participated in the study. In parallel to the original SHIP-START study, a new independent sample was drawn to conduct research of similar scope (SHIP-TREND). The data used in our analyses were derived from this SHIP-TREND cohort, which was initiated 10 years after SHIP-START in the same region. A total of 4420 individuals participated in the SHIP-TREND study. From this pool of participants, 1264 underwent polysomnography (PSG) examination. All participants gave written informed consent. The final samples consisted of 216 and 186 participants, respectively (see also *Characteristics of our study cohort* and *Statistical analysis*). The study was approved by the ethics committee of the University Medicine Greifswald and complies with the Declaration of Helsinki. Data used in our analyses originates from baseline examinations, which took place between 2008 and 2012 (SHIP-TREND-0). A detailed description of the assessment of all included variables can be found in the Supplementary Materials.

### Characteristics of our study cohort

#### Inclusion and exclusion criteria

**Fig. 1:**
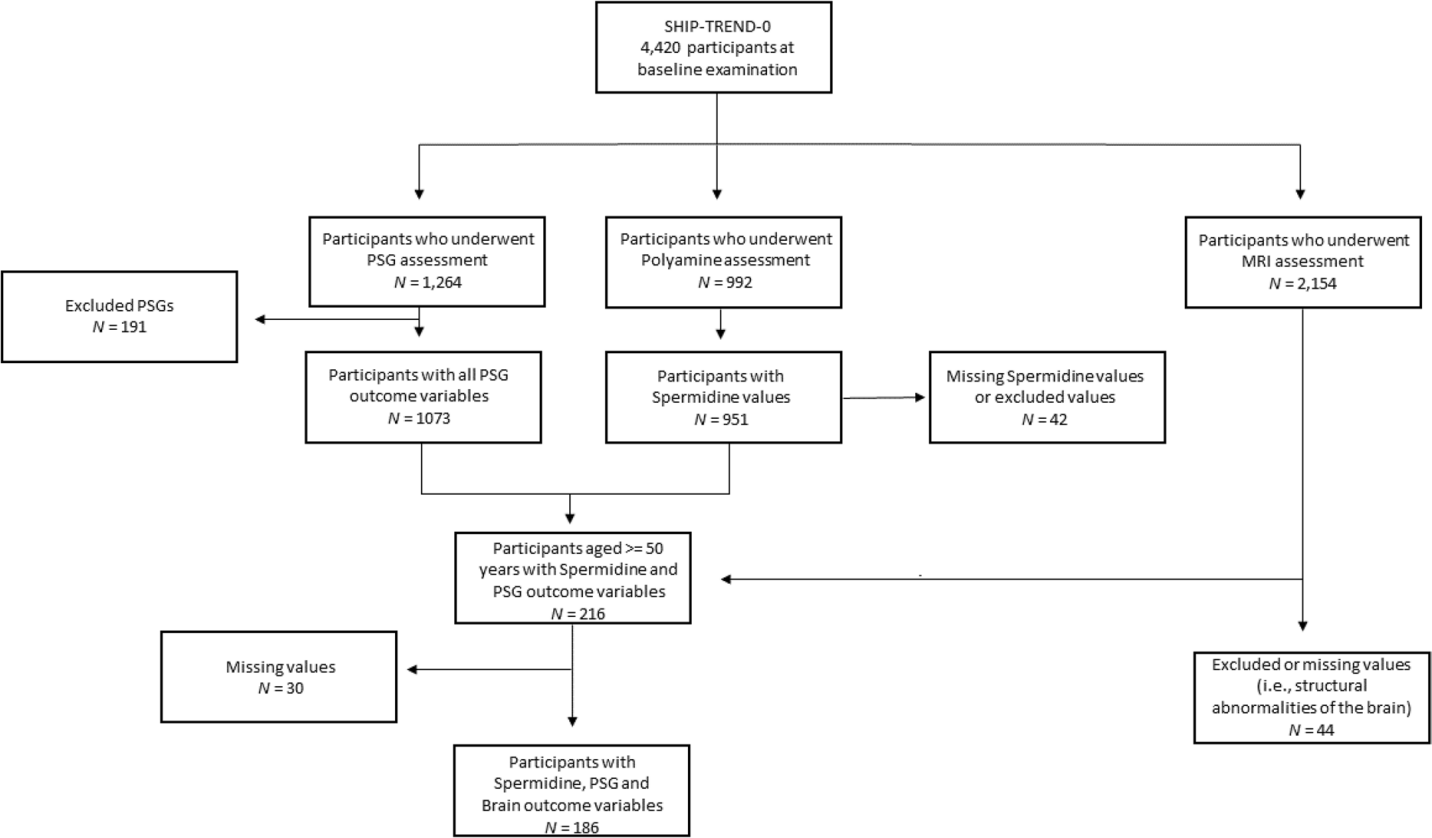
Flow chart showing the selection of the study sample

From the original 4420 individuals who took part in the SHIP-TREND study, all participants who underwent polyamine (*N* = 992), PSG (*N* = 1264), or MRI (*N* = 2154) assessment were initially included into the study. In a second step, only participants who were aged 50 years or older were included into the study, since we were particularly interested in the associations between spermidine plasma levels, sleep, and brain health in aging. Participants with spermidine plasma values exceeding ± 3 SD of the mean plasma concentration were excluded from the study sample (*N* = 2). Additionally, PSGs that were shorter than 2 h or longer than 10 h were excluded (*N* = 191) leading to a sample of 216 participants with spermidine and PSG outcome variables. Participants with structural abnormalities of the brain (e.g., tumors, cysts, ventricular dilatations; *N* = 44) or missing values (*N* = 30) were also excluded, leading to a sample of 186 participants with spermidine, PSG, and brain outcome variables (see Fig. 1 for a flowchart showing the selection of the study sample).

#### Magnetic resonance imaging

T1-weighted and fluid-attenuated inversion recovery (FLAIR) images of the head were obtained from the same 1.5 T scanner (Siemens Magnetom Avanto scanner; Siemens, Erlangen, Germany). The following measuring parameters were used: T1: orientation = axial plane, repetition time (TR) = 1900 ms, echo time (TE) = 3.37 ms, flip angle = 15°, slice thickness = 1 mm, and resolution = 1 mm × 1 mm and FLAIR: orientation = axial plane, TR = 5000 ms, TE = 325 ms, slice thickness = 3 mm, and resolution = 0.9 mm × 0.9 mm. For details, please see also [32].

### Alzheimer’s disease score

This score is based on a classifier that differentiates individuals with AD from cognitively healthy ones [15]. Briefly, L2-penalized (ridge) logistic regression was used to train a binary classifier using T1-weighted MRI scans of 374 individuals, including AD patients (*N* = 165) and healthy controls (*N* = 209), from the Alzheimer’s Disease Neuroimaging Initiative (ADNI) 1 case control cohort [33]. Cortical reconstruction and volumetric segmentation were performed with FreeSurfer, and 169 volumetric features of GM, white matter, and the ventricular system were used to optimally separate individuals with AD from cognitively healthy ones [34]. The continuous AD score is defined as the linear predictor of the logistic model, i.e., log(*p*/(1 − *p*)) with *p* denoting the probability of having AD. This means a higher score indicates a higher similarity of the subject’s brain to an AD brain as defined by the ADNI-1 sample. The classifier was validated in an independent data set (*N* = 416) of the Open Access Series of Imaging Studies [35]. For more details, see Frenzel et al. [36].

### Statistical analysis

Statistical analysis was performed using R version 3.6.1 [37]. For multivariable regression analyses, we used the “lm” function for fitting ordinary least squares regression models [37]. Since we were particularly interested in the associations between spermidine plasma levels, sleep, and brain health in aging, we restricted our analysis to participants aged 50 years or older.

Because we were exploring the link between sleep, spermidine plasma levels and brain aging, we were particularly interested in parameters of sleep macroarchitecture and microarchitecture that were previously associated with AD pathology [7, 8, 10, 19]. These included SWS duration, spectral power in the SO (< 1 Hz) and spindle frequency range (12–16 Hz), and SO-spindle coupling assessed by coupling strength as well as spindle activity during SO upstate.

In a first step, these electroencephalogram (EEG) markers of sleep were used as outcome variables to evaluate the association of the exposure variable spermidine and SWS physiology. This data set comprised 216 participants.

In a second step, a magnetic resonance imaging (MRI) marker of brain health operationalized as AD score was used as outcome variable to examine the association between the exposure variable spermidine and AD score. We were interested in demonstrating that elevated spermidine plasma levels were associated with deteriorated brain health, similar to findings from a larger subsample of 659 individuals across the lifespan (i.e., 21 to 81 years) of the SHIP-TREND cohort [26] in this smaller SHIP-TREND cohort, which included only adult participants aged 50 to 81 years, who took part in the PSG assessment. In a third step, the association between the five EEG markers of sleep as exposure variables and brain health as outcome variable were assessed.

Finally, a mediation analysis was performed to evaluate if the association between spermidine blood levels and AD score is mediated by markers of SWS physiology. Mediation analysis was performed with the help of the R package “mediate” [38]; the mediation and direct effect, as well as their 95% CIs, were estimated via bootstrapping with 100,000 simulations each. For these last three steps, the dataset comprised 186 participants.

All models, including the mediation analysis, were adjusted for age and sex. The AD score was additionally adjusted for intracranial volume with the help of the ratio method (i.e., proportion approach [39]). All regression models including parameters of sleep microarchitecture were performed separately for frontal (F3, F4) and central (C3, C4) regions of interest (see also Supplementary Materials*Sleep macroarchitecture and microarchitecture*). Finally, to reach normal distribution, a log transformation was applied to spermidine, SO power, and spindle power. We used false discovery rate (FDR) to control for multiple testing [40]. Results were considered statistically significant at a *q* value of < 0.05. In both datasets, participants with missing data on one of the variables of interest were excluded from analysis.

## Results

### Descriptive statistics

Descriptive characteristics of the study sample are shown in Table 1. Our sample for the first step of our analysis comprised 216 participants (mean age 60.64 years ± 7.17 years, 120 women). The sample for our last two analysis steps included 186 participants (mean age 60.51 years ± 7.25 years, 104 women).

**Table 1:** Descriptive characteristics of study sample

Characteristics	Mean (SD) or *N* (%)
Sociodemographic parameters	
Female, *N* (%)	120 (55.6)
Age (year)	60.64 (± 7.17)
Education (year)	13.35 (± 2.58)
Sleep macroarchitecture	
Time in bed (min)	462.09 (± 46.77)
Sleep efficiency (%)	87.6 (± 8.5)
Sleep duration (min)	303.92 (± 75.04)
Duration of N1 (min)	14.34 (± 9.06)
Duration of N2 (min)	159.74 (± 63.10)
Duration of N3 (SWS) (min)	25.46 (± 20.62)
Duration of REM (min)	34.35 (± 20.77)
Sleep fragmentation (number of times/h)	2.73 (± 0.87)
Sleep microarchitecture	
Frontal SO power (μV^2^)	188.21 (± 160.17)
Central SO power (μV^2^)	134.56 (± 109.34)
Frontal spindle power (μV^2^)	4.88 (± 4.81)
Central spindle power (μV^2^)	5.74 (± 3.50)
Frontal spindle activity during SO upstate (μV^2^)	0.47 (± 0.16)
Central spindle activity during SO upstate (μV^2^)	0.56 (± 0.16)
Frontal coupling strength	0.93 (± 0.01)
Central coupling strength	0.94 (± 0.01)
Polyamines	
Spermidine (μM)	0.125 (± 0.052)
Brain imaging measures	
Alzheimer’s disease score^a^	− 4.076 (± 1.293)
Intracranial volume (L)^a^	1.56 (0.15)
Lifestyle	
Alcohol consumption (ethanol in g/day)^b,c^	9.01 (± 11.86)
Smoking status, *N* (%)	
Non-smoker	100 (46.3)
Previous smoker	99 (45.8)
Smoker	17 (7.9)
Waist-to-hip ratio	0.89 (± 0.084)
BMI (kg/m^2^)	28.61 (± 4.35)

Values are presented as mean and standard deviation or *N* and %. Unless indicated differently *N* = 216

*BMI* body mass index

^a^*N* = 186

^b^*N* = 214

^c^Alcohol consumption was calculated as alcohol consumption during the last 30 days

### Association between spermidine and SWS physiology

Analyses on the relationship between spermidine plasma levels and the five different markers of sleep revealed two significant associations between spermidine and coupling between SO and spindle activity (Table 2).

**Table 2:** Association between spermidine and sleep parameters of interest

	Spermidine	
	*ß* (95% CI)	*q* value
SWS duration		
	− 0.086 (− 0.21, 0.05)	0.269
SO power		
Frontal	− 0.028 (− 0.16, 0.10)	0.905
Central	− 0.079 (− 0.21, 0.05)	0.326
Spindle power		
Frontal	0.001 (− 0.13, 0.13)	0.999
Central	− 0.096 (− 0.22, 0.03)	0.186
Spindle activity during SO upstate		
Frontal	− 0.177 (− 0.30, − 0.04)	0.015
Central	− 0.134 (− 0.26, 0.01)	0.050
Coupling strength		
Frontal	− 0.057 (− 0.19, 0.07)	0.619^ꝉ^
Central	− 0.181 (− 0.31, − 0.05)	0.014

*ß*: standardized regression coefficient; 95% CI with lower and upper bound; *q*: *q* value. A log transformation was applied to spermidine, spindle power, and SO power. All models were adjusted for age and sex. Detailed results for all regression models including all variables can be found in the Supplementary Materials (Tables S1-S9)

*SWS* slow-wave sleep, *SO* slow oscillations, *CI* confidence interval

^ꝉ^This regression model does not explain variance

The regression model evaluating the association between spermidine and frontal spindle activity during SO upstate explained 14% of the variance [*R*^*2*^ = 0.14, adj*. R*^2^ = 0.12, *F*(3, 212) = 11.15] and revealed an inverse association between spermidine and spindle activity during SO upstate (*ß* =  − 0.177, *q* = 0.015). Finally, the regression model evaluating the association between spermidine and central coupling strength explained 12% of the variance [*R*^2^ = 0.12, adj*. R*^*2*^ = 0.11, *F*(3, 212) = 9.73] and revealed an inverse association between spermidine and spindle activity during SO upstate (*ß* =  − 0.181, *q* = 0.014). This indicates that elevated spermidine levels were significantly associated with lower coupling between slow oscillations and spindle activity. All other regression models were not significant (all *q*_*s*_ ≥ 0.05).

### Association between spermidine and brain health

The regression model on the association between spermidine and AD score explained 19% of the variance [*R*^2^ = 0.18, adj. *R*^2^ = 0.17, *F*(3, 182) = 13.69] and revealed a positive association (*ß* = 0.172, *q* = 0.023), indicating that participants with elevated spermidine plasma levels were more likely to have AD-related brain pathology (Table 3, see also [26]).

**Table 3:** Association between spermidine and AD score

	Spermidine	
	*ß* (95% CI)	*q* value
AD score		
	0.175 (0.03, 0.31)	0.016

*ß*: standardized regression coefficient; 95% CI with lower and upper bound; *q*: *q* value. A log transformation was applied to spermidine. Model was adjusted for age and sex. AD score was adjusted for intracranial volume. Detailed results for the regression model including all variables can be found in the Supplementary Materials (Table S10)

*AD* Alzheimer’s disease, *CI* confidence interval

### Association between brain health and SWS physiology

Analyses on the relationship between AD score and the five different sleep markers of interest revealed no significant associations (all *q*_*s*_ ≥ 0.05, Table 4).

**Table 4:** Association between AD score and sleep parameters of interest

	AD score	
	*ß* (95% CI)	*q* value
SWS duration		
	0.057 (− 0.08, 0.19)	0.562
SO power		
Frontal	0.076 (− 0.06, 0.21)	0.364
Central	− 0.013 (− 0.02, 0.25)	0.150
Spindle power		
Frontal	− 0.028 (− 0.16, 0.10)	0.916
Central	− 0.007 (− 0.15, 0.13)	0.999
Spindle activity during SO upstate		
Frontal	− 0.073 (− 0.21, 0.06)	0.413
Central	− 0.030 (− 0.17, 0.11)	0.915
Coupling strength		
Frontal	− 0.097 (− 0.23, 0.03)	0.209
Central	− 0.027 (− 0.16, 0.11)	0.936

*ß*: standardized regression coefficient; 95% CI with lower and upper bound; *q*: *q* value. A log transformation was applied to spermidine, SO power, and spindle power. Model was adjusted for age and sex. AD score was adjusted for intracranial volume. Detailed results for all regression models including all variables can be found in the Supplementary Materials (Table S11-S19)

*AD* Alzheimer’s disease, *SWS* slow-wave sleep, *SO* slow oscillations, *CI* confidence interval

### Mediation analysis

Finally, we were interested if coupling between slow oscillations and spindle activity would mediate the association between spermidine plasma levels and AD score, since spermidine plasma levels were significantly associated with spindle activity during SO upstate and coupling strength. Results show that neither frontal spindle activity during SO upstate nor central coupling strength mediated the significant association between spermidine plasma levels and brain health operationalized as AD score (Tables 5 and 6).

**Table 5:** Overview of the mediation analysis testing the mediation effect of frontal spindle activity during SO upstate on the significant association between spermidine plasma levels and AD score

	Spermidine	
AD score	Estimate [95% CI]	*p* value
Average causal mediation effect	1.75E^−08^ [− 5.74E^−08^, 0.00]	0.65
Average direct effect	3.84E^−07^ [7.86E^−08^, 0.00]	0.016*
Total effect	4.02E^−07^ [9.10E^−08^, 0.00]	0.013*
Proportional effect	4.35E^−02^ [− 1.82E^−01^, 0.29]	0.65

95% CI with lower and upper bound

*AD* Alzheimer’s disease, *CI* confidence interval, *SO* slow oscillation

*p value < 0.05; **p value < 0.01

**Table 6:** Overview of the mediation analysis testing the mediation effect of central coupling strength on the significant association between spermidine plasma levels and AD score

	Spermidine	
AD score	Estimate [95% CI]	*p* value
Average causal mediation effect	− 1.83E^−09^ [− 8.89E^−08^, 0.00]	0.93
Average direct effect	4.04E^−07^ [9.39E^−08^, 0.00]	0.013*
Total effect	4.02E^−07^ [9.02E^−08^, 0.00]	0.014*
Proportional effect	− 4.55E^−03^ [− 2.95E^−01^, 0.19]	0.92

95% CI with lower and upper bound

*AD* Alzheimer’s disease, *CI* confidence interval

*p value < 0.05; **p value < 0.0

## Discussion

The aim of the present study was to evaluate the interrelations of spermidine levels, SWS physiology, and brain health in aging using a community-based cross-sectional sample. We found that higher spermidine levels were associated with lower coupling between slow oscillations and spindle activity (i.e., lower frontal spindle activity during SO upstate and lower central coupling strength). SWS duration, SO power, and spindle power showed no significant associations with spermidine. In addition, in this subsample of 186 individuals, aged 50 to 81 years, we demonstrated that elevated spermidine plasma levels were associated with deteriorated brain health operationalized as AD score, similar to findings in the larger subsample of 659 individuals, aged 21 to 81 years, from the same SHIP-TREND study [26]. Sleep parameters of interest were not associated with AD score. Finally, there was no mediation effect of coupling between slow oscillations and spindle activity for the association between elevated spermidine levels and brain health.

### Spermidine and sleep physiology

The significant associations between higher spermidine plasma levels and less advantageous microparameters of sleep, in particular spindle activity during SO upstate and coupling strength, seem to contradict studies in cell cultures and animal models [27–29]. In particular, Zwighaft et al. [27] demonstrated that spermidine can control the circadian period in cell cultures and mice by modulating the interrelations between the clock proteins Per2 and Cry1. In addition, the authors showed that age-related decrease in spermidine was associated with prolonged circadian periods especially in aging mice. Finally, they demonstrated that spermidine supplementation was not only able to reverse longer circadian periods in aging mice, but that spermidine deficiency could also increase circadian period length in young mice. Similarly, Huang et al. [29] showed that spermidine supplementation could restore age-related accumulation of presynaptic active zone vesicles that lead to memory impairment in *Drosophila* [41], and reverse adverse changes in sleep physiology during aging, in particular, suppress age-associated sleep pattern changes in daytime and night-time sleep. Interestingly, Bedont et al. [42] found increased polyamine levels and concomitant reduced nitrogen levels in *Drosophila* mutants with impaired sleep. While supplementation of polyamines prolonged sleep in control flies, it was toxic for mutants with impaired sleep. These findings suggest that polyamine accumulation and nitrogen stress may be possible mechanisms of disturbed sleep physiology, consistent with our results showing that elevated spermidine blood levels were associated with less advantageous microsleep physiology and thus sleep quality.

### The association of spermidine and SO-spindle coupling

Moreover, spermidine plasma levels showed significant associations with SO-spindle coupling. SO-spindle coupling strength was previously demonstrated to be uniquely and specifically associated with early tauopathy in the human medial temporal lobe (MTL [19]). Additionally, recent animal studies indicate a complex relationship between tau pathology and polyamine dysregulation in mice [43, 44]. Pathological tau accumulation in mice elicited chronic activation of the polyamine-stress response leading to increased polyamine production and their acetylated counterparts [43]. Importantly, this overproduction of polyamines and their acetylation in turn aggravated tau pathology in mice with underlying tauopathy, supporting a feed-forward cycle of disease progression [44]. Thus, the link between spermidine and sleep may be mediated via the association between spermidine levels and tau burden, with tau-related decrease of structural integrity in the MTL impacting on coupling strength between SO and spindles [19]. SO-spindle coupling during NREM sleep requires intact communication between different brain regions, including the medial temporal cortex, but most importantly medial prefrontal cortex (mPFC) and thalamus [45]. However, while structural grey matter volume in the mPFC is also strongly associated with SO activity during NREM sleep [46, 47], tau deposition in this region is not typical in healthy older adults or early preclinical stages of AD [48–50], which may also explain the lack of association between spermidine and SO power in our study.

Beyond the link to tau burden and spermidine level, SO-spindle coupling during sleep plays a functional role in memory consolidation [51]. With regard to age-related changes, several studies showed that SO-spindle coupling is compromised in older adults and paralleled by impaired overnight memory consolidation [52–54]. A therapeutic approach using sleep modulation and early spermidine supplementation could therefore act on multiple levels to prevent disease progression by interrupting the feed-forward cycle of disease progression while also improving memory.

### Parameters of sleep microstructure and AD score

Finally, parameters of sleep microstructure and AD score were not significantly associated. This finding was unexpected, given that Weihs et al. [55] demonstrated an inverse association of less slow-wave sleep with higher AD scores in a SHIP-TREND subsample including 712 individuals. One possibility for this lack of association could be that our subsample, which included only 186 individuals, did not have enough power to show a significant association. Alternatively, AD score as a general measure for brain health might not be sensitive enough for associations with specific parameters of sleep microstructure. Recent studies (for a review, see Mander [49]) could show that especially slow wave activity was associated with mPFC/anterior cingulate cortex (ACC) and posterior cingulate cortex (PCC)/precuneus, whereas coupling between SO and sleep spindle activity was associated with MTL.

### Strengths and limitations

A major strength of this population-based study is the uniqueness and completeness of the available data, which not only provided extensive sociodemographic information but also included physiological spermidine blood levels, MRI data, and PSG parameters. These characteristics have allowed us to investigate for the first time the relationship between spermidine plasma levels, brain health, and microparameters of sleep within a comparatively large and healthy human population. Second, SHIP-TREND provides both high-quality standards and a rigorous standardization of all methods used [51]. Third, detailed assessments of not only sleep stages but also fine-grained parameters of sleep microstructure allowed us to investigate sleep parameters previously associated with AD pathophysiology [44].

Several limitations should be considered when interpreting our results. First, the possibility of a selection bias must be considered since a large number of participants refused or were not able to participate in the MRI and PSG assessment. Nevertheless, in total data on both, MRI and PSG were available from around 701 participants which allowed us to draw representative conclusions from our study. Second, during PSG data collection, the site of the PSG assessment had to change twice. However, these changes were documented in detail and no influence on the data quality could be detected by the responsible PSG assessors [52]. Third, due to resource constraints, only a single PSG night was recorded in the sleep laboratory [52]. This could lead to potential biases affecting data quality (i.e., the effect of the first night). However, this was a necessary limitation to perform a large number of PSG recordings. In addition, a review of the distribution of sleep stages for our cohort revealed a similar distribution in the range of previously reported studies [53]. Fourth, because this study is cross-sectional, it is not possible to draw causal conclusions. Nonetheless, to best of our knowledge, our study provides first important insights into the relationships between elevated spermidine plasma levels, brain health, and microparameters of sleep. Finally, in this study, we were interested in five key parameters of microsleep that have previously been associated with the accumulation of Aß and tau, which are both neuropathological hallmarks of AD. However, Aß and tau accumulation was not measured in the current study. Future studies need to evaluate whether sleep physiology, spermidine, and Aß/tau deposition are interrelated and if sleep may play a mediating role in these potential associations. Additionally, based on our results, it should be investigated if SO-spindle coupling is associated with more specific parameters of AD including tau and Aß burden in MTL and mPFC/ACC, respectively, as determined by positron emission tomography (PET).

## Conclusions and clinical implications

In sum, we could show for the first time that higher spermidine plasma levels were associated with lower coupling between slow oscillations and spindle activity (i.e., spindle activity during SO upstate and coupling strength) in a cross-sectional cohort of older individuals. Although these inverse associations do not reflect beneficial effects of spermidine supplementation on sleep quality found in animal models, they conceptually agree with findings from post-mortem and human cohort studies that demonstrated an association of higher spermidine tissue or blood levels with deteriorated brain health. These findings support the notion that elevated spermidine blood levels could serve as a potential biomarker for AD. Findings from supplementation studies of spermidine in humans are still ambiguous, while most animal studies report beneficial effects on brain function and health. Potentially, the positive effects of spermidine supplementation on age-related deficits in sleep physiology in animal models could open strategies to prevent or even treat functional and structural deterioration of the aging brain. Thus, future supplementation studies should be coupled with detailed assessment of macrostructure as well as microstructure of sleep in older adults, and ideally include tau and beta amyloid biomarkers, to shed further light on these questions.

### Supplementary Information

Below is the link to the electronic supplementary material.

Supplementary file1 (DOCX 51 KB)

## Data Availability

Data used in in this article was obtained from “SHIP – Study of Health in Pomerania”. Requests to access the data set may be sent to “Forschungsverbund Community Medicine” (community-medicine@uni-greifswald.de).
