# Supplementary material for: Association of spermidine blood levels with microstructure of sleep—implications from a population-based study
Source: GeroScience. 2023 Aug 7;46(1):1319–30. doi: 10.1007/s11357-023-00886-3 (PMC10828152; doi:10.1007/s11357-023-00886-3)
Supplement: Supplementary file 1 — Supplementary file1 (DOCX 51 KB) [file 11357_2023_886_MOESM1_ESM.docx]

Supplementary Material

**Polyamine assessment**

Spermidine and spermine plasma levels were assessed via the AbsoluteIDQ p180 Kit (Biocrates Life Sciences AG, Innsbruck, Austria) at the Institute of Clinical Chemistry and Laboratory Medicine of the University Medicine Greifswald, Germany. First, 10 µL aliquots of each plasma sample were processed in a fully automated assay as recommended by the manufacturer. Spermidine and spermine were detected by using the LC–MS/MS method with an Agilent C18 column. MS analyses were performed on an AB SCIEX 5500 QTrap™ mass spectrometer (AB SCIEX, Darmstadt, Germany) with electrospray ionization combined with an HPLC system (Agilent 1260 Infinity Binary LC, Santa Clara, CA, USA). Data were preprocessed with Analyst software (Version 1.5.1, AB SCIEX, Darmstadt, Germany) including peak integration and concentration determination from calibration curves. Preprocessed data were uploaded into the Biocrates MetIDQ software, and the metabolite concentrations were automatically calculated. To account for day-to-day variation in performance of the LC-MS/MS platform, a unique sample dependent normalization was performed. To this end, for each plate the measured concentrations of the metabolites were divided by the median concentration leading to equal median values for each metabolite on each plate. Subsequently, the median of the plate medians was calculated to reset to the original scale (μM concentrations).

**Socio-Demographic Variables**

Sociodemographic factors and medical history were assessed by a computer-assisted face-to-face- interview. Participant’s years of education were assessed by interview. The years of education were calculated from the years of schooling (i.e., secondary school, high school) and vocational training (i.e., vocational school diploma, university degree), considering the heterogeneity of the education system in Germany and former East Germany. Smoking was defined as current smoking (occasional; 1–14 cigarette(s) per day; >15 cigarettes per day), former smoking (occasional; 1–14 cigarette(s) per day; >15 cigarettes per day) and never smoking. Alcohol consumption of the last 30 days prior assessment was calculated in g per day. The interview was followed by the medical examination which included the measurement of height and weight to calculate the body mass index as well as the measurement of waist circumference in cm to calculate waist-to-hip ratio.

**Sleep assessment and sleep scoring**

Participants underwent a single‐night, laboratory‐based polysomnographic (PSG) examination (Alice 5 System, Philips Respironics, Eindhoven, The Netherlands) which was performed by specifically certified personnel according
to the American Academy of Sleep Medicine (AASM) guide-
lines. Participants were permitted to choose their own bedtime and to sleep for as long as they wished. For reliability reasons, however, a minimal bedtime of around 8 hours was required. For details on the PSG study protocol see also Stubbe et al. (2016).

Electroencephalogram (EEG) assessment included 6 channels of interest (F3, F4, C3, C4, O1, O2), each referenced to the contralateral mastoid. Additionally, electrooculogram, electromyogram (chin and tibialis muscles) and electrocardiogram were recorded according to standard sleep monitoring. EEG signals were filtered between 0.32 and 106 Hz and sampled at a rate of 2000 Hz.

Sleep staging was performed automatically with the Greifswald Sleep Stage Classifier [2], which is based on a deep learning approach. Scoring accuracy of the algorithm was 88.8 % with the 5-expert consensus of the DREEM Open Dataset [3]. Staging was calculated using the consensus of the F3, F4, C3 and C4 EEG channels and the EOG1, EOG2, and HEOG (subtraction of EOG1 and EOG2) channels. To avoid anomalous data on the edges of the PSG, the first and last 10 minutes were cut from each PSG before sleep staging. Every 30-sec epoch was scored as either wake, NREM sleep stage 1 (N1), 2 (N2), 3 (N3) or REM sleep.

**Sleep macro- and micro-architecture**

For sleep macro-architecture, time spent in different sleep stages, sleep duration, sleep efficiency (sleep duration divided by time in bed) and sleep fragmentation were evaluated. For more details, please see Table S1. EEG preprocessing for assessment of sleep microstructure first included referencing to a more standard average mastoid reference, in which the M1-M2 channel derivation was used to re-reference with the following formula:

For each channel c, the re-referenced channel r is:

r = c + (M1-M2) / 2, if c is referenced to M2 and

r = c - (M1-M2) / 2, if c is referenced to M1.

Data were then filtered with a notch filter at 50Hz for line noise, and a finite impulse response bandpass of 0.3-30Hz. Bad channels were algorithmically marked with the ANOAR package ([4]; see Appendix), which identifies channels that do not correlate with neighboring channels for prolonged periods. EMG and other aberrant sections of data were identified and excluded by dividing the raw data into sections of 500ms. Any section which contained a sample-to-sample jump of more than 100 microvolts, or where the difference between the maximum and minimum voltage was more than 500 microvolts was marked as noisy and excluded from further analysis. The following analyses were performed separately for frontal (F3, F4) and central (C3, C4) regions using only artifact-free data from N2 and N3.

**Sleep outcome variables**

*SWS duration:*

SWS duration was determined as duration of sleep stage N3 (in minutes).

*Spectral power analysis*:

Spectral power was calculated for each participant across all N2/N3 sections of sleep in the SO and Spindle band (0.5-1.25Hz; 12-16Hz). Power spectral density was calculated using Welch’s method [5], and the band power was the integral of the PSD curve within that the SO frequency band. This was done separately for each 30s epoch within the N2/N3 sections, averaged across epochs.

*SO-spindle coupling:*

To assess the temporal coupling of SO and spindles, we employed two complementary analytic event-locked approaches: time-frequency representation (TFR) analysis and the synchronization index (SI) procedure. TFR is a relatively indirect way of measuring the relationship between SO phase and spindle activity (in comparison to SI), as because it offers insights about the time-power relationship of SO-spindle coupling. The SI, by contrast, provides a more fine-grained analysis of the phase-power relationship between the phase of the SO and power of spindle activity, but is also a complex-valued, circular variable, which significantly constrains statistical analysis. We therefore conducted both complementary analysis and compared results.

For both approaches, we first identified SO events according to an established algorithm [6,7]. EEG signal was first bandpass filtered at 0.16-1.25Hz, and every two successive positive-to-negative zero-crossings that had a minimum of 0.8s and maximum of 2s between them were considered potential SO candidates. Trough to peak amplitude of all candidates was measured, and all candidates in the 75th percentile were considered SOs.

*TFR (spindle activity during SO upstate)*: TFR decomposes the EEG signal into a time-resolved power spectrum within a given range of frequencies, in our case this is the typical frequency range of spindles (12-16 Hz). We apply TFR decomposition to each SO; because SOs have a relatively uniform phase in their frequency band (0.5-1.25Hz), a consistent presence of spindle band power in the TFR decomposition of SOs is an indirect indication of phase-amplitude coupling. TFR was performed with a morlet wavelet decomposition of 5 cycles on the 5-20Hz band, on the -2 to 1.5 second time period centered around the trough of the SO. The first and last 100ms were cut to remove edge effects, and a z-score method baseline of -1.9 to -1 seconds was subtracted from the TFR.

As previous evidence indicates that mean sleep spindle activity preferably occurred during or close to the SO up-state [7,8] we extracted individual Spindle power values (frequency range 13-17 Hz) from the time window 300-600ms after SO trough for correlation analysis with spermidine plasma levels as well as structural brain scores.

*SI (coupling strength)*: The SI procedure explores the spindle dynamics in more detail, as it measures the phase of the SOs where spindle band power reached its maximum and quantifies synchronicity between fluctuations in spindle band power and the SO phase [9,10].

$$\mathrm{SI}=\frac{1}{m}\sum_{j=1}^{m} e^{i[ϴ_{\mathrm{SO}}(t_{j}) - ϴ_{\mathrm{sp}}(t_{j})]}$$

where m is the number of samples in an epoch, θ_SO_ is the instantaneous phase in the slow oscillation band, θ_sp_ is the instantaneous phase of the power fluctuations in the spindle band, and t_j_ is the EEG voltage at time index j. Power and instantaneous phase estimation were estimated with the wavelet method as implemented in the Python package Tensorpac (0.6.5) [11].

SI is a complex number, the radius (absolute value) of which (SIr) shows the strength of coupling between SO and spindle amplitude in our analyses. The SO-spindle coupling strength was calculated for each participant.

**I. Association between Spermidine and SWS physiology**

*Association between Spermidine and SWS duration*

**Table S1:** Results of the association between spermidine and SWS duration as outcome variable

| Variable | ß | 95% CI | *q* |
| --- | --- | --- | --- |
| Spermidine^a^ | -0.086 | -0.21, 0.05 | .269 |
| Age | -0.137 | -0.26, -0.01 | .074 |
| Sex | -0.231 | 0.09, 0.36 | .003 |

ß: standardized regression coefficient; 95% CI with lower and upper bound; *q:* q-value. ^a^ A log transformation was applied to spermidine. *N* = 216. Abbreviations: SWS, slow-wave sleep; CI, confidence interval.

*Association between Spermidine and frontal SO Power*

**Table S2:** Results of the association between spermidine and frontal SO power^b^ as outcome variable

| Variable | ß | 95% CI | *q* |
| --- | --- | --- | --- |
| Spermidine^a^ | -0.028 | -0.16, 0.10 | .905 |
| Age | -0.171 | -0.30, -0.03 | .047 |
| Sex | 0.118 | -0.01, 0.25 | .179 |

ß: standardized regression coefficient; 95% CI with lower and upper bound; *q:* q-value. ^a^ A log transformation was applied to spermidine. ^b^ A log transformation was applied to SO power. *N* = 216. Abbreviations: SO, slow oscillations; CI, confidence interval.

*Association between Spermidine and central SO Power*

**Table S3:** Results of the association between spermidine and central SO power^b^ as outcome variable

| Variable | ß | 95% CI | *q* |
| --- | --- | --- | --- |
| Spermidine^a^ | -0.079 | -0.21, 0.05 | .326 |
| Age | -0.126 | -0.25, 0.00 | .112 |
| Sex | 0.227 | 0.09, 0.36 | .004 |

ß: standardized regression coefficient; 95% CI with lower and upper bound; *q:* q-value. ^a^ A log transformation was applied to spermidine. ^b^ A log transformation was applied to SO power. *N* = 216. Abbreviations: SO, slow oscillations; CI, confidence interval.

*Association between Spermidine and frontal Spindle Power*

**Table S4:** Results of the association between spermidine and frontal spindle power^c^ as outcome variable

| Variable | ß | 95% CI | *q* |
| --- | --- | --- | --- |
| Spermidine^a^ | 0.001 | -0.13, 0.13 | 0.999 |
| Age | -0.252 | -0.38, -0.12 | 0.001 |
| Sex | 0.047 | -0.08, 0.18 | 0.976 |

ß: standardized regression coefficient; 95% CI with lower and upper bound; *q:* q-value. ^a^ A log transformation was applied to spermidine. ^c^ A log transformation was applied to spindle power. *N* = 216. Abbreviations: CI, confidence interval.

*Association between Spermidine and central Spindle Power*

**Table S5:** Results of the association between spermidine and central spindle power^c^ as outcome variable

| Variable | ß | 95% CI | *q* |
| --- | --- | --- | --- |
| Spermidine^a^ | -0.096 | -0.22, 0.03 | .186 |
| Age | -0.268 | -0.39, -0.14 | .000 |
| Sex | 0.234 | 0.10, 0.36 | .001 |

ß: standardized regression coefficient; 95% CI with lower and upper bound; *q:* q-value. ^a^ A log transformation was applied to spermidine. ^c^ A log transformation was applied to spindle power. *N* = 216. Abbreviations: CI, confidence interval.

*Association between Spermidine and frontal Spindle activity during SO upstate*

**Table S6:** Results of the association between spermidine and frontal spindle activity during SO upstate as outcome variable

| Variable | ß | 95% CI | *q* |
| --- | --- | --- | --- |
| Spermidine^a^ | -0.177 | -0.30, -0.04 | .015 |
| Age | -0.283 | -0.40, -0.15 | .000 |
| Sex | 0.115 | -0.01, 0.24 | .106 |

ß: standardized regression coefficient; 95% CI with lower and upper bound; *q:* q-value. ^a^ A log transformation was applied to spermidine. *N* = 216. Abbreviations: SO, slow oscillations; CI, confidence interval.

*Association between Spermidine and central Spindle activity during SO upstate*

**Table S7:** Results of the association between spermidine and central spindle activity during SO upstate as outcome variable

| Variable | ß | 95% CI | *q* |
| --- | --- | --- | --- |
| Spermidine^a^ | -0.134 | -0.26, 0.01 | .050 |
| Age | -0.351 | -0.47, -0.22 | .000 |
| Sex | 0.163 | 0.03, 0.28 | .023 |

ß: standardized regression coefficient; 95% CI with lower and upper bound; *q:* q-value. ^a^ A log transformation was applied to spermidine. *N* = 216. Abbreviations: SO, slow oscillations; CI, confidence interval.

*Association between Spermidine and frontal Coupling Strength*

**Table S8:** Results of the association between spermidine and frontal coupling strength as outcome variable

| Variable | ß | 95% CI | *q* |
| --- | --- | --- | --- |
| Spermidine^a^ | -0.057 | -0.19, 0.07 | .619^ꝉ^ |
| Age | -0.169 | -0.30, -0.03 | .619 |
| Sex | -0.051 | -0.18, 0.08 | .051 |

ß: standardized regression coefficient; 95% CI with lower and upper bound; *q:* q-value. ^a^ A log transformation was applied to spermidine. *N* = 216. Abbreviations: CI, confidence interval. ^ꝉ^This regression model does not explain variance.

*Association between Spermidine and central Coupling Strength*

**Table S9:** Results of the association between spermidine and central coupling strength as outcome variable

| Variable | ß | 95% CI | *q* |
| --- | --- | --- | --- |
| Spermidine^a^ | -0.181 | -0.31, -0.05 | .014 |
| Age | -0.292 | -0.41, -0.16 | .000 |
| Sex | 0.023 | -0.10, 0.15 | .966 |

ß: standardized regression coefficient; 95% CI with lower and upper bound; *q:* q-value. ^a^ A log transformation was applied to spermidine. *N* = 216. Abbreviations: CI, confidence interval.

**II. Association between Spermidine and Brain health**

*Association between Spermidine and AD Score*

**Table S10:** Results of the association between spermidine and AD score as outcome variable

| Variable | ß | 95% CI | *q* |
| --- | --- | --- | --- |
| Spermidine^a^ | 0.175 | 0.03, 0.31 | .016 |
| Age | 0.306 | 0.17, 0.43 | .000 |
| Sex | -0.211 | -0.34, -0.07 | .005 |

ß: standardized regression coefficient; 95% CI with lower and upper bound; *q:* q-value. ^a^ A log transformation was applied to spermidine. *N* = 186. Abbreviations: AD, Alzheimer’s Disease; CI, confidence interval.

**III. Association between Brain health and SWS physiology**

*Association between SWS duration and AD Score*

**Table S11:** Results of the association between SWS duration and AD score as outcome variable

| Variable | ß | 95% CI | *q* |
| --- | --- | --- | --- |
| SWS | 0.057 | -0.08, 0.19 | .562 |
| Age | 0.309 | 0.17, 0.44 | .000 |
| Sex | -0.271 | -0.40, -0.13 | .000 |

ß: standardized regression coefficient; 95% CI with lower and upper bound; *q:* q-value. *N* = 186. Abbreviations: AD, Alzheimer’s Disease; CI, confidence interval.

*Association between frontal SO Power and AD Score*

**Table S12:** Results of the association between frontal SO power and AD score as outcome variable

| Variable | ß | 95% CI | *q* |
| --- | --- | --- | --- |
| frontal SO Power^b^ | 0.076 | -0.06, 0.21 | .364 |
| Age | -0.267 | -0.40, -0.13 | .000 |
| Sex | 0.311 | 0.17, 0.44 | .000 |

ß: standardized regression coefficient; 95% CI with lower and upper bound; *q:* q-value. ^b^ A log transformation was applied to SO power. *N* = 186. Abbreviations: SO, slow oscillations; AD, Alzheimer’s Disease; CI, confidence interval.

*Association between central SO Power and AD Score*

**Table S13:** Results of the association between central SO power and AD score as outcome variable

| Variable | ß | 95% CI | *q* |
| --- | --- | --- | --- |
| central SO Power^b^ | -0.013 | -0.02, 0.25 | .150 |
| Age | 0.313 | 0.17, 0.44 | .000 |
| Sex | -0.287 | -0.42, -0.14 | .000 |

ß: standardized regression coefficient; 95% CI with lower and upper bound; *q:* q-value. ^b^ A log transformation was applied to SO power. *N* = 186. Abbreviations: SO, slow oscillations; AD, Alzheimer’s Disease; CI, confidence interval.

*Association between frontal Spindle Power and AD Score*

**Table S14:** Results of the association between frontal spindle power and AD score as outcome variable

| Variable | ß | 95% CI | *q* |
| --- | --- | --- | --- |
| frontal Spindle Power^c^ | -0.028 | -0.16, 0.10 | .916 |
| Age | -0.253 | 0.15, 0.43 | .000 |
| Sex | 0.293 | -0.38, -0.11 | .001 |

ß: standardized regression coefficient; 95% CI with lower and upper bound; *q:* q-value. ^c^ A log transformation was applied to spindle power. *N* = 186. Abbreviations: AD, Alzheimer’s Disease; CI, confidence interval.

*Association between central Spindle Power and AD Score*

**Table S15:** Results of the association between central spindle power and AD score as outcome variable

| Variable | ß | 95% CI | *q* |
| --- | --- | --- | --- |
| central Spindle Power^c^ | -0.007 | -0.15, 0.13 | .999 |
| Age | 0.297 | 0.15, 0.43 | .000 |
| Sex | -0.253 | -0.39, -0.11 | .001 |

ß: standardized regression coefficient; 95% CI with lower and upper bound; *q:* q-value. ^c^ A log transformation was applied to spindle power. *N* = 186. Abbreviations: AD, Alzheimer’s Disease; CI, confidence interval.

*Association between frontal Spindle activity during SO upstate and AD Score*

**Table S16:** Results of the association between frontal spindle activity during SO upstate and AD score as outcome variable

| Variable | ß | 95% CI | *q* |
| --- | --- | --- | --- |
| frontal Spindle activity during SO upstate | -0.073 | -0.21, 0.06 | .413 |
| Age | 0.278 | 0.13, 0.41 | .000 |
| Sex | -0.242 | -0.37, -0.10 | .001 |

ß: standardized regression coefficient; 95% CI with lower and upper bound; *q:* q-value. *N* = 186. Abbreviations: SO, slow oscillations; AD, Alzheimer’s Disease; CI, confidence interval.

*Association between central Spindle activity during SO upstate and AD Score*

**Table S17:** Results of the association between central spindle activity during SO upstate and AD score as outcome variable

| Variable | ß | 95% CI | *q* |
| --- | --- | --- | --- |
| central Spindle activity during SO upstate | -0.030 | -0.17, 0.11 | .915 |
| Age | 0.288 | 0.14, 0.43 | .000 |
| Sex | -0.248 | -0.38, -0.11 | .001 |

ß: standardized regression coefficient; 95% CI with lower and upper bound; *q:* q-value. *N* = 186. Abbreviations: SO, slow oscillations; AD, Alzheimer’s Disease; CI, confidence interval.

*Association between frontal Coupling Strength and AD Score*

**Table S18:** Results of the association between frontal coupling strength and AD score as outcome variable

| Variable | ß | 95% CI | *q* |
| --- | --- | --- | --- |
| frontal coupling strength | -0.097 | -0.23, 0.03 | .209 |
| Age | 0.285 | 0.15, 0.42 | .000 |
| Sex | -0.257 | -0.39, -0.12 | .000 |

ß: standardized regression coefficient; 95% CI with lower and upper bound; *q:* q-value. *N* = 186. Abbreviations: AD, Alzheimer’s Disease; CI, confidence interval.

*Association between central Coupling Strength and AD Score*

**Table S19:** Results of the association between central coupling strength and AD score as outcome variable

| Variable | ß | 95% CI | *q* |
| --- | --- | --- | --- |
| central coupling strength | -0.027 | -0.16, 0.11 | .936 |
| Age | 0.292 | 0.15, 0.43 | .000 |
| Sex | -0.252 | -0.38, -0.11 | .001 |

ß: standardized regression coefficient; 95% CI with lower and upper bound; *q:* q-value. *N* = 186. Abbreviations: AD, Alzheimer’s Disease; CI, confidence interval.

**References**

1. Stubbe B, Penzel T, Fietze I *et al.* Polysomnography in a large population based study—the Study of Health in Pomerania protocol. *J Sleep Disord Manag* 2016;**2**:1–5.

2. Hanna J, Flöel A. An accessible and versatile deep learning-based sleep stage classifier . *Front Neuroinformatics*  2023;**17**.

3. Guillot A, Sauvet F, During EH *et al.* Dreem open datasets: Multi-scored sleep datasets to compare human and automated sleep staging. *IEEE Trans neural Syst Rehabil Eng* 2020;**28**:1955–65.

4. Hanna J, Pulvermüller F. Congruency of Separable Affix Verb Combinations Is Linearly Indexed by the N400. *Front Hum Neurosci* 2018;**12**:219.

5. Welch P. The use of fast Fourier transform for the estimation of power spectra: a method based on time averaging over short, modified periodograms. *IEEE Trans audio Electroacoust* 1967;**15**:70–3.

6. Mölle M, Bergmann TO, Marshall L *et al.* Fast and slow spindles during the sleep slow oscillation: disparate coalescence and engagement in memory processing. *Sleep* 2011;**34**:1411–21.

7. Mölle M, Marshall L, Gais S *et al.* Grouping of spindle activity during slow oscillations in human non-rapid eye movement sleep. *J Neurosci* 2002;**22**:10941–7.

8. Klinzing JG, Mölle M, Weber F *et al.* Spindle activity phase-locked to sleep slow oscillations. *Neuroimage* 2016;**134**:607–16.

9. Staresina BP, Bergmann TO, Bonnefond M *et al.* Hierarchical nesting of slow oscillations, spindles and ripples in the human hippocampus during sleep. *Nat Neurosci* 2015;**18**:1679–86.

10. Ladenbauer J, Ladenbauer J, Külzow N *et al.* Promoting sleep oscillations and their functional coupling by transcranial stimulation enhances memory consolidation in mild cognitive impairment. *J Neurosci* 2017;**37**:7111–24.

11. Combrisson E, Nest T, Brovelli A *et al.* Tensorpac: an open-source Python toolbox for tensor-based Phase-Amplitude Coupling measurement in electrophysiological brain signals. *PLoS Comput Biol* 2020;**16**:e1008302.
